# Supplementary material for: Changes in child suicide rates and characteristics during the COVID-19 pandemic in England
Source: Front Child Adolesc Psychiatry. 2026 Jun 16;5:1657552. doi: 10.3389/frcha.2026.1657552 (PMC13314999; doi:10.3389/frcha.2026.1657552)
Supplement: Supplementary file 1 [file Supplementaryfile1.docx]

**Appendix 1. Sensitivity Analysis#1: Different time windows for heraldic events (n=275)**

| **Characteristic** | **Total** | **Odds Ratio (95% confidence interval)** | | |
| --- | --- | --- | --- | --- |
|  |  | **Conditional Regression** | **Random Effects (Unadjusted)** | **Random Effect (Adjusted)*** |
| **2-9 days** | | | | |
| **Deaths Linked** | 275 |  |  |  |
| **Self-Harm** |  | 21.89 (6.40-74.82) | 18.17 (6.06-54.51) | 18.56 (6.17-55.86) |
| **Eating Disorder** |  | 4.00 (0.25-63.95) | 4.01 (0.25-64.37) | 4.04 (0.25-65.50) |
| **Anxiety Disorder** |  | 9.83 (0.99-97.23) | 6.06 (1.01-36.47) | 6.13 (1.01-37.07) |
| **PTSD** |  | NA | NA | NA |
| **Mood Disorder** |  | NA | NA | NA |
| **Schizophrenia** |  | NA | NA | NA |
| **Any of above** |  | 16.19 (6.64-39.46) | 12.79 (5.91-27.67) | 13.03 (6.01-28.29) |
| **2-90 days** | | | | |
| **Deaths Linked** | 275 |  |  |  |
| **Self-Harm** |  | 6.38 (3.06-12.28) | 3.72 (2.14- 6.48) | 3.80 (2.17-6.65) |
| **Eating Disorder** |  | 0.45 (0.08-2.44) | 0.57 (0.13-2.52) | 0.56 (0.13-2.52 |
| **Anxiety Disorder** |  | 2.40 (0.77-7.45) | 2.02 (0.75-5.45) | 2.03 (0.75-5.48) |
| **PTSD** |  | NA | NA | NA |
| **Mood Disorder** |  | 0.94 (0.32-2.80) | 0.95 (0.36-2.55) | 0.95 (0.35-2.56) |
| **Schizophrenia** |  | NA | NA | NA |
| **Any of above** |  | 5.41 (2.74-10.69) | 2.16 (1.44-3.25) | 2.19 (1.45-3.30) |

Values are OR (95% CI)

NA – Not Available

**Appendix 2. Sensitivity Analysis#2: Changes in characteristics of childhood by HES linkage (n=497)**

| **Characteristic** | **n** |  | **HES-not Linked** | **HES-Linked** | **p-value** |
| --- | --- | --- | --- | --- | --- |
| **Total deaths** |  |  | **222** | **275** |  |
| **Year of Death (April to March)** | 497 |  |  |  | <0.001 |
| **2019** |  |  | 17 (7.7%) | 94 (34.2%) |  |
| **2020** |  |  | 23 (10.4%) | 101 (36.7%) |  |
| **2021** |  |  | 64 (28.3%) | 80 (29.1%) |  |
| **2022** |  |  | 118 (53.2%) | 0 (0.0%) |  |
| **Sex** | 496 |  |  |  | 0.412 |
| **Female** |  |  | 82 (36.9%) | 105 (38.3%) |  |
| **Male** |  |  | 140 (63.1%) | 169 (61.7%) |  |
| **Race/Ethnicity** | 474 |  |  |  | 0.013 |
| **Asian or Asian British** |  |  | 23 (10.7%) | 10 (3.9%) |  |
| **Black or Black British** |  |  | 8 (3.7%) | 12 (4.6%) |  |
| **Mixed** |  |  | 20 (9.3%) | 18 (7.0%) |  |
| **Other** |  |  | 9 (4.2%) | 5 (1.9%) |  |
| **White** |  |  | 155 (72.1%) | 214 (82.6%) |  |
| **Age (Years)** |  |  |  |  | 0.894 |
| **5-15** |  |  | 99 (44.6%) | 121 (44.0%) |  |
| **15-17** |  |  | 123 (55.4%) | 154 (56.0%) |  |
| **Deprivation Quintile** | 493 |  |  |  | 0.488 |
| **1 (most deprived)** |  |  | 48 (21.9%) | 59 (21.5%) |  |
| **2** |  |  | 41 (18.7%) | 56 (20.4%) |  |
| **3** |  |  | 45 (20.6%) | 60 (21.9%) |  |
| **4** |  |  | 34 (15.5%) | 52 (19.0%) |  |
| **5 (least deprived)** |  |  | 51 (23.3%) | 47 (17.2%) |  |
| **Region** | 497 |  |  |  | 0.636 |
| **East Midlands** |  |  | 20 (9.0%) | 29 (10.6%) |  |
| **East of England** |  |  | 26 (11.7%) | 34 (12.4%) |  |
| **London** |  |  | 33 (14.9%) | 27 (9.8%) |  |
| **North East** |  |  | 9 (4.1%) | 14 (5.1%) |  |
| **North West** |  |  | 32 (14.4%) | 32 (11.6%) |  |
| **South East** |  |  | 32 (14.4%) | 51 (18.6%) |  |
| **South West** |  |  | 23 (10.4%) | 33 (12.0% |  |
| **West Midlands** |  |  | 24 (10.8%) | 32 (11.6%) |  |
| **Yorkshire and Humber** |  |  | 23 (10.4%) | 23 (8.4%) |  |
| **LGBT+** | 497 |  | 25 (11.3%) | 34 (12.4%) | 0.706 |
| **Not trans** |  |  | 13 (5.9%) | 17 (6.2%) | 0.879 |
| **Trans** |  |  | 12 (5.4%) | 17 (6.2%) | 0.714 |
| **Method** |  |  |  |  | 0.002 |
| **Fall or fracture** |  |  | 17 (7.7%) | 25 (9.1%) |  |
| **Firearms** |  |  | NA | NA |  |
| **Hanging or Strangulation** |  |  | 136 (61.3%) | 207 (75.3%) |  |
| **Jumping or lying in front of a moving object** |  |  | 37 (16.7%) | 27 (9.8%) |  |
| **Other** |  |  | NA | NA |  |
| **Poisoning** |  |  | 22 (9.9%) | 13 (0.4%) |  |
| **Unclear** |  |  | NA | NA |  |

Values are n(%)

NA – Not Available
